# Association between dementia and hepatitis B and C virus infection

**DOI:** 10.1097/MD.0000000000026476

**Published:** 2021-07-23

**Authors:** Hyo Geun Choi, Jae Seung Soh, Jae Sung Lim, Song Yong Sim, Suk Woo Lee

**Affiliations:** a Department of Otorhinolaryngology-Head & Neck Surgery, Hallym University College of Medicine, Anyang, Republic of Korea; b Hallym Data Science Laboratory, Hallym University College of Medicine, Anyang, Republic of Korea; c Department of Internal Medicine, Hallym University Sacred Heart Hospital, Hallym University College of Medicine, Anyang, Republic of Korea; d Department of Neurology, Hallym University Sacred Heart Hospital, Hallym University College of Medicine, Anyang, Republic of Korea; e Department of Statistics and Institute of Statistics, Hallym University, Chuncheon, Republic of Korea; f Department of Obstetrics and Gynecology, Hallym University Sacred Heart Hospital, Hallym University College of Medicine, Anyang, Republic of Korea.

**Keywords:** cohort study, dementia, epidemiology, hepatitis virus infection, nested case–control study

## Abstract

Several viral infections are known to increase the risk of dementia through brain cell damage and systemic infection. The association between hepatitis B and C virus (HBV and HCV) infections and dementia was evaluated using a national sample cohort from South Korea. Using the national cohort study from the Korean National Health Insurance Service, we extracted data for patients with HBV or HCV infection and for matched control participants. The controls were matched to the patients according to age, sex, income, region of residence, and past medical histories. The incidence of HCV infection was higher in the dementia group (1.0% [113/11,228]) than in the control group (0.8% [364/44,912], *P* = .043). However, there was no difference in the incidence of HBV infection in the dementia and control groups. The adjusted odds ratio (OR) for HCV infection was 1.25 (95% confidence interval [CI] = 1.01–1.54, *P* = .043) in the dementia group. According to the subgroup analysis by sex, the adjusted ORs for HCV infection were 1.04 (95% CI = 072–1.49, *P* = .851) in men and 1.38 (95% CI = 1.06–1.79, *P* = .016) in women. We concluded that the incidence of HCV infection was higher (with a higher OR) in women with dementia than in matched control participants in South Korea.

## 1 Introduction

Dementia is a gradual and progressive decline in cognitive function that interferes with the ability to function independently.^[1]^ The clinical characteristics of dementia include memory loss, communication and language impairment, agnosia, apraxia, and impaired executive function.^[2]^ The main pathogenesis of dementia includes abnormal deposits of amyloid beta and hyperphosphorylation of tau protein in the hippocampus and cortex; these deposits destroy synapses that mediate memory and cognition.^[3]^ Previous studies have shown that viral, fungal, or bacterial infection can be involved in the pathogenesis of dementia by involving inflammatory pathways and causing neuroinflammation in dementia.^[4,5]^

Chronic hepatitis is mainly caused by the hepatitis B and C viruses (HBV/HCV) and can lead to both acute and chronic hepatitis, ranging in severity from a mild illness to a serious, lifelong illness including liver cirrhosis and hepatocellular carcinoma.^[6,7]^ HBV and HCV are also associated with extrahepatic manifestations, including renal, cardiovascular, metabolic, systemic inflammatory, and neurologic disorders.^[8–10]^ Neuropsychiatric disorders, including cognitive impairment, depression, anxiety, and fatigue, have been reported in chronic HBV or HCV infections, although neurological symptoms are more common in HCV infection than in HBV infection.^[11,12]^ These neurological manifestations of viral hepatitis occur irrespective of the severity of hepatitis or hepatic encephalopathy.^[12]^

Studies on the effects of HCV infection on cognitive dysfunction have been conducted since the early 2000s, and recently, several large-scale cohort studies of the effects of HCV infection on cognitive function have been reported.^[13]^ In a nationwide study in Taiwan, the adjusted hazard ratio (HR) for dementia was 1.36 (95% confidence interval [CI], 1.27–1.42) for the HCV population.^[14]^ In another nationwide study of Taiwan, patients with Alzheimer disease showed a 2.31-fold (95% CI = 1.28–4.16) increased risk of HCV infection.^[15]^ However, these cohort studies have been conducted in very few races, and few studies have been reported, making the results insufficient to generalize conclusions about the association between HCV infection and dementia.

The aim of this study was to investigate the risk of dementia in patients with HBV and HCV in South Korea using a nationwide, population-based dataset obtained from the Korean National Health Insurance Service).

## 2 Materials and methods

### 2.1 Study population and data collection

This national cohort study was conducted using the Korean Health Insurance Review and Assessment Service National Sample Cohort. For a detailed explanation of these data, refer to previous studies.^[16,17]^ This study was approved by the ethics committee of Hallym University (2019-01-003). Written informed consent was waived by the Institutional Review Board.

### 2.2 Participant selection

Out of 1,125,691 cases with 114,369,638 medical claim codes, we included cases of participants who were diagnosed with dementia from 2002 through 2013 (n = 13,102). Dementia was categorized if the participants were diagnosed with Alzheimer disease (international classification of diseases, 10th revision [ICD-10] codes: G30) or dementia in Alzheimer disease (F00). For the accuracy of diagnosis, we only selected participants who were treated ≥2 times. We describe the reliability of the diagnosis of dementia in Supplementary material S1, Supplemental Digital Content. The control participants were extracted from 1,112,589 participants who were never diagnosed with dementia from 2002 through 2013 among this cohort.

Hepatitis infection was included if the participant was diagnosed using ICD-10 codes: chronic viral hepatitis B with delta agent (B18.0); chronic viral hepatitis B without delta agent (B18.1); and chronic viral hepatitis C (B18.2). Finally, a total of 38,691 hepatitis B and 7584 hepatitis C participants were included.

**Figure 1:**
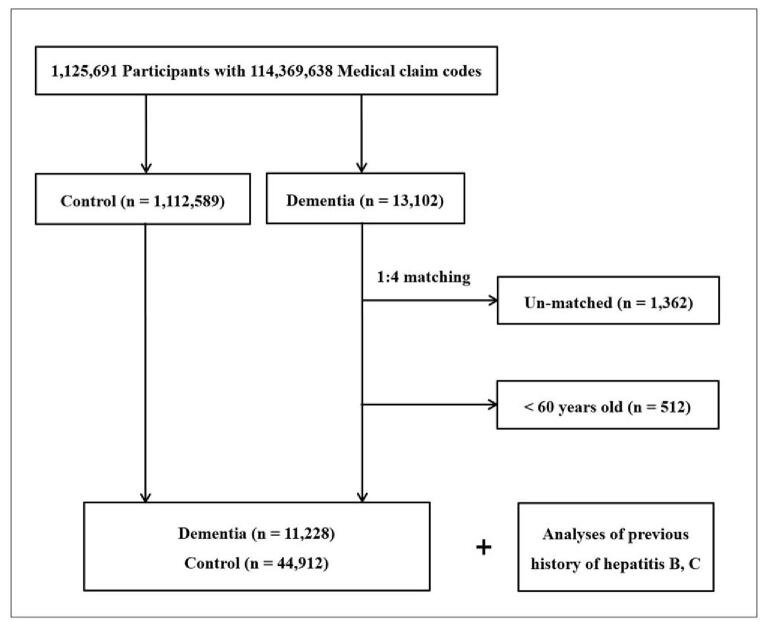
A schematic illustration of the participant selection process that was used in the present study. Out of a total of 1,125,691 participants, 11,228 dementia participants were matched with 44,912 control participants for age, group, sex, income group, region of residence, and past medical histories.

We matched participants who were diagnosed with dementia at a 1:4 ratio with participants (control group) who were not diagnosed with dementia from 2002 through 2013 (Fig. 1). The controls were selected from the general population (n = 1,112,589). The matches were processed for age, group, sex, income group, region of residence, and past medical histories (hypertension, diabetes mellitus [DM], and dyslipidemia). To prevent selection bias when selecting matched participants, the control group participants were sorted using a random number order and then selected from top to bottom. The matched control participants were assumed to be involved simultaneously with each matched patient who was diagnosed with dementia (index date). Therefore, control participants who died before the index date were excluded. The dementia participants for whom we could not identify a sufficient number of matching participants were excluded (n = 1362). We also excluded participants under 60 years old (n = 512). Finally, 1:4 matching resulted in the inclusion of 11,228 dementia participants and 44,912 control participants. After matching, we analyzed the previous histories of hepatitis B and C in both the dementia and control groups.

### 2.3 Variables

The age groups were classified using 5-year intervals: 60–64, 65–69, 70–74…, and 85+ years old. A total of 6 age groups were designated. The income groups were initially divided into 41 classes (1 health aid class, 20 self-employment health insurance classes, and 20 employment health insurance classes). These groups were reclassified into 11 classes (class 1 [lowest income] to 11 [highest income]). Region of residence was divided into 16 areas according to the administrative district. These regions were regrouped into urban (Seoul, Busan, Daegu, Incheon, Gwangju, Daejeon, and Ulsan) and rural (Gyeonggi, Gangwon, Chungcheongbuk, Chungcheongnam, Jeollabuk, Jeollanam, Gyeongsangbuk, Gyeongsangnam, and Jeju) areas.

The past medical histories of the participants were evaluated using ICD-10 codes. For the accuracy of diagnosis, hypertension (I10 and I15), DM (E10-E14), and dyslipidemia (E78) were assessed if the participants were treated ≥2 times.

### 2.4 Statistical analyses

Chi-square tests were used to compare the general characteristics between the dementia and control groups.

Conditional logistic regression analysis was used to analyze the odds ratio (OR) for hepatitis associated with dementia. In this analysis, crude (simple) and adjusted (age, sex, income, region of residence, hypertension, DM, and dyslipidemia) models were used, and the 95% CIs were calculated.

For the subgroup analyses, we divided the participants by sex (men and women)

To analyze the OR linking hepatitis with dementia, logistic regression analysis was used. In this analysis, crude (simple) and adjusted (age, sex, income, region of residence, hypertension, diabetes, dyslipidemia histories) models were used. The 95% CI was calculated.

Two-tailed analyses were conducted, and *P* values <.05 were considered to indicate significance. The results were analyzed using SPSS v. 22.0 (IBM, Armonk, NY, USA).

## 3 Results

The general characteristics (age, sex, income, region of residence, hypertension, DM, and dyslipidemia) of the participants were identical due to matching (*P* = 1.000). The rate of HCV infection was higher in the dementia group (1.0% [113/11,228]) than in the control group (0.8% [364/44,912], *P* = .043, Table 1). There was no statistically significant difference in the incidence of HBV infection between the dementia and control groups (1.6% [177/11,228] vs 1.4% [628/44,912]; *P* = .156; Table 1).

**Table 1:** General characteristics of participants.

	Total participants		
Characteristics	Dementia (n, %)	Control group (n, %)	*P*
Age (years old)			1.000
60–64	580 (5.2)	2320 (5.2)	
65–69	1288 (11.5)	5152 (11.5)	
70–74	2321 (20.7)	9284 (20.7)	
75–79	2965 (26.4)	11860 (26.4)	
80–84	2604 (23.2)	10416 (23.2)	
85+	1470 (13.1)	5880 (13.1)	
Sex			1.000
Male	3573 (31.8)	14,292 (31.8)	
Female	7655 (68.2)	30,620 (68.2)	
Income			1.000
1 (lowest)	1282 (11.4)	5128 (11.4)	
2	1087 (9.7)	4348 (9.7)	
3	446 (4.0)	1,784,446 (4.0)	
4	479 (4.3)	1916 (4.3)	
5	517 (4.6)	2068 (4.6)	
6	620 (5.5)	2480 (5.5)	
7	712 (6.3)	2848 (6.3)	
8	773 (6.9)	3092 (6.9)	
9	1065 (9.5)	4260 (9.5)	
10	1726 (15.4)	6904 (15.4)	
11 (highest)	2521 (22.5)	10,084 (22.5)	
Region of residence			1.000
Urban	4502 (40.1)	18,008 (40.1)	
Rural	6726 (59.9)	26,904 (59.9)	
Hypertension			1.000
Yes	8199 (73.0)	32,796 73.0)	
No	3029 (27.0)	12,116 (27.0)	
Diabetes mellitus			1.000
Yes	3948 (35.2)	15,792 (35.2)	
No	7280 (64.8)	29,120 (64.8)	
Dyslipidemia			1.000
Yes	3473 (30.9)	13,892 (30.9)	
No	7755 (69.1)	31,020 (69.1)	
Hepatitis B			.156
Yes	177 (1.6)	628 (1.4)	
No	11,051 (98.4)	44,284 (98.6)	
Hepatitis C			.043^∗^
Yes	113 (1.0)	364 (0.8)	
No	11,115 (99.0)	44,548 (99.2)	

∗ Chi-square test or Fisher exact test. Significance at *P* < .05.

The adjusted ORs for HBV and HCV infection were 1.13 (95% CI = 0.96–1.34, *P* = .155) and 1.25 (95% CI = 1.01–1.54, *P* = .043) in the dementia group, respectively (Table 2).

**Table 2:** Crude and adjusted odds ratios (95% confidence interval) of hepatitis B and C infection for dementia.

	Odds ratios			
Characteristics	Crude	*P*	Adjusted^†^	*P*
Hepatitis B	1.13 (0.96–1.34)	0.156	1.13 (0.96–1.34)	.155
Control	1.00		1.00	
Hepatitis C	1.24 (1.01–1.54)	0.043^∗^	1.25 (1.01–1.54)	.043^∗^
Control	1.00		1.00	

∗ Conditional logistic regression analyses. Significance at *P* < .05.

† Adjusted model for age, sex, income, region of residence, hypertension, diabetes mellitus, and dyslipidemia histories.

According to the subgroup analysis by sex, the adjusted ORs for HCV infection were 1.04 (95% CI = 0.72–1.49, *P* = .851) in men and 1.38 (95% CI = 1.06–1.79, *P* = .016) in women (Table 3).

**Table 3:** Subgroup analyses of crude and adjusted odds ratios (95% confidence interval) of hepatitis C infection for dementia according to sex.

	ORs of dementia			
Characteristics	Crude	*P*	Adjusted^†^	*P*
Men (n = 17,865)				
Hepatitis C	1.04 (0.72–1.49)	0.851	1.04 (0.72–1.49)	.851
Control	1.00		1.00	
Women (n = 38,275)				
Hepatitis C	1.38 (1.06–1.79)	0.016∗	1.38 (1.06–1.79)	.016^∗^
Control	1.00		1.00	

∗ Conditional logistic regression analyses, significance at *P* < .05.

† Adjusted model for age, sex, income, region of residence, hypertension, diabetes mellitus, and dyslipidemia histories.

## 4 Discussion

In the present nationwide cohort study, the incidence of HCV infection was higher in patients with dementia, yielding a higher OR than that in matched control participants, especially in women. However, there was no difference in the incidence of HBV infection between the dementia group and the control group.

There are various causes of dementia: cerebrovascular disease, genetic factors, toxic, or metabolic disorders that cause extensive brain cell damage, physical activity, smoking, educational level, and alcohol consumption.^[18]^ However, the possibility that viral or bacterial infections, including those caused by herpes virus, human immunodeficiency virus, *Chlamydia pneumoniae*, and spirochete bacteria, are the etiologies in the pathogenesis of dementia is attracting increasing interest.^[19–23]^

Hepatic encephalopathy is a neurological dysfunction of the brain that results from severe hepatic failure, and hepatitis-related neurological dysfunctions are frequently observed mainly at cirrhotic or pre-cirrhotic stages.^[24]^ However, studies have reported that cognitive dysfunction in patients with chronic hepatitis precedes the process of cirrhosis and is not related to disease severity in the liver. Forton et al first evaluated the role of HCV in causing cerebral function abnormalities. They used proton magnetic resonance spectroscopy to determine that compared with a control group, patients with HCV infection showed cerebral metabolic abnormalities (elevated choline/creatinine ratio) in the basal ganglia and white matter.^[13]^ In the following year, the same group reported significant impairments in concentration and working memory in HCV-infected patients compared to the control group.^[25]^ Since these original investigations, several large-scale studies have been performed to evaluate cognitive dysfunction in patients with HCV infection. In a nationwide study in Taiwan, HCV infection increased the risk for dementia independent of conventional risk factors (HR 1.36, 95% CI = 1.27–1.42).^[14]^ In another nationwide study, patients with Alzheimer disease showed a 2.31-fold (95% CI = 1.28–4.16) increased risk of hepatitis C infection adjusted for demographics and socioeconomic status.^[15]^ Research on the relationship between HBV infection and cognitive impairment is limited, and comparative studies of a small number of subjects have been reported.^[11]^

The mechanisms by which HCV infection increases the risk of cognitive impairment are not well understood, but 2 hypotheses have been introduced. First, HCV can cross the blood–brain barrier and replicate not only within hepatocytes but also within the central nervous system and has a direct toxic effect on brain cells in patients with cognitive impairment. HCV-positive patients show neuronal impairment within the white matter, cortical hypoperfusion, and hyperperfusion in the basal ganglia, which indicates brain inflammation resulting in cerebral dysfunction evaluable by magnetic resonance spectroscopy.^[26]^ In another study, the HCV sequence and replicative form were detected in microglia/macrophages of autopsied brain tissue and cerebrospinal fluid from infected patients.^[27]^ Fletcher et al detected functional HCV receptors in brain cells using neuroepithelioma cell lines and reported that HCV may infect cells of the central nervous system.^[28]^ In their follow-up study, HCV replication and receptors were detected in brain microvascular endothelial and brain endothelial cells, and they concluded that brain endothelial cell lines support HCV entry and replication by compromising blood–brain barrier integrity.^[29]^ The expression of HCV receptors on microvascular endothelial cells in the brain is different from that occurring during HBV infection. Second, systemic inflammation has an important role in the pathogenesis of dementia.^[30]^ Inflammatory cytokines such as interleukins (IL)-1β, IL-6, IL-10, IL-12, and tumor necrosis factor-α and the resulting systemic inflammation affect the levels of unfavorable biomarkers of dementia by increasing the burden of amyloid beta-peptide and tau protein and decreasing the hippocampal volume.^[31]^ The liver has a central role in regulating systemic inflammation and hepatic HCV infection and in activating immune reactions and systemic inflammatory cytokines, which lead to extrahepatic manifestations, including cognitive impairment.^[32]^ An immune-phenotyping study reported that compared with HBV infection, infection with HCV yields a unique pattern of immune alteration. This finding suggests a potential mechanism by which HCV infection shows a greater tendency toward neurologic symptoms than HBV infection does.^[33]^

Contrary to the above results, several studies have shown that hepatitis is not associated with cognitive disorders. Hilsabeck et al reported that there is no different pattern of cognitive deficits between patients with chronic hepatitis C and those with chronic liver disease of different etiologies.^[34]^ Abrantes et al found no evidence of an association between HCV infection and cognitive impairment when applying strict criteria such as depression disorder.^[35]^ However, the above studies showed limitations in that they were performed in a small number of subjects and were conducted as cross-sectional studies.

Our investigation showed that the OR for dementia was higher in the HCV group than in the control group in women but not in men. HCV infection increases with age and is more prevalent in women than men, and there is a difference in the prevalence of dementia between men and women; women have a higher risk of developing dementia than men.^[36,37]^ These sexual differences in developing dementia were assumed to be differences in genetic susceptibility, brain anatomy, brain function, and the social and behavioral environment.^[38]^ In a longitudinal, clinicopathologic cohort study, women had a more global Alzheimer disease pathology, such as more neurofibrillary tangles and neuritic plaques.^[39]^ HCV infection was proven to be positively correlated with cerebral microglial activation, and microglial activation was also positively correlated with tau and amyloid deposition in the cerebral cortex in an in vivo study using positron emission tomography.^[40,41]^ Considering the results of the above study, it can be hypothesized that if women who are dementia patients have more severe brain lesions than their male counterparts, then the toxic effects of hepatitis C virus on brain cells would significantly affect the risk of dementia in women but not in men. More research is needed on the risks and the differences in the mechanism of dementia according to sex.

This study has several limitations. First, dementia and HBV/HCV were diagnosed according to the ICD codes from the administrative claims data and were based on a count of the number of visits for dementia and HBV/HCV, which may not have been reflective of the actual number of dementia or HBV/HCV incidents experienced by the patients. Using ICD codes from big claim-code data could carry the possibility of misdiagnosis. Second, although the control group was matched for medical histories of several conditions as well as for demographic factors, some possible confounders were not considered, including alcohol intake, smoking, body mass index, depressive disorder, and education level. Third, depending on the severity of HBV/HCV, various neurological symptoms can occur. However, this study does not reflect the severity of HBV/HCV infection.

There were several strengths of this investigation. First, we used a population-based dataset consisting of 1 million subjects with a 12-year follow-up period to assess the risk of dementia in patients with HBV/HCV infection. Our study is the first to evaluate the association between HBV/HCV infection and dementia in a nationwide cohort study in South Korea. Second, the control group was matched with the HBV/HCV infection group not only for basic characteristics, including age, sex, income, and region of residence but also for risk factors for dementia, such as hypertension, DM, and dyslipidemia. This detailed matching might provide valid evidence for the effect of HBV/HCV infection on dementia.

## 5 Conclusion

We concluded that women with dementia had a higher incidence of HCV infection than women without dementia in South Korea.

## Author contributions

**Conceptualization:** Hyo Geun Choi.

**Data curation:** Hyo Geun Choi, Song Yong Sim.

**Formal analysis:** Hyo Geun Choi, Jae Seung Soh, Song Yong Sim.

**Funding acquisition:** Hyo Geun Choi.

**Investigation:** Jae Sung Lim, Suk Woo Lee.

**Methodology:** Hyo Geun Choi.

**Project administration:** Hyo Geun Choi, Suk Woo Lee.

**Resources:** Jae Sung Lim.

**Software:** Hyo Geun Choi, Jae Seung Soh, Jae Sung Lim, Song Yong Sim.

**Supervision:** Hyo Geun Choi, Jae Seung Soh, Jae Sung Lim, Song Yong Sim.

**Validation:** Jae Seung Soh, Jae Sung Lim, Suk Woo Lee.

**Visualization:** Jae Seung Soh, Jae Sung Lim, Suk Woo Lee.

**Writing – original draft:** Suk Woo Lee.

**Writing – review & editing:** Suk Woo Lee.

## Supplementary Material

Supplemental Digital Content
