# Supplementary material for: Association between dementia and hepatitis B and C virus infection
Source: Medicine (Baltimore). 2021 Jul 23;100(29):e26476. doi: 10.1097/MD.0000000000026476 (PMC8294892; doi:10.1097/MD.0000000000026476)
Supplement: Supplemental Digital Content [file medi-100-e26476-s001.docx]

**Supplemental material 1 (S1) Description of diagnosis of dementia**

Dementia was categorized if the participants were diagnosed Alzheimer's disease (G30) or Dementia in Alzheimer's disease (F00). We selected if the participants were treated ≥ 2 times.

In this national sample cohort, 123,025 participants were ≥ 65 years old in 2012 year. Among them, 9,740 (7.9%) of participants were categorized as dementia according to our methods (5.4% [n =2,758] in male; 9.7% [n= 6,982] in female).

We could compare these results of central dementia center of Korea ([www.nid.or.kr](http://www.nid.or.kr)) which is controlled by Ministry of Health and Welfare of Korea. The earliest data was 2012 year, and it was available in ≥ 65 years old. According to their data, the prevalence of dementia (Alzheimer's disease, and others) except vascular dementia were 7.63% (4.47% in male; 9.85% in female).
